# Genomic inbreeding and population structure of northern pike (*Esox lucius*) in Xinjiang, China

**DOI:** 10.1002/ece3.7469

**Published:** 2021-04-06

**Authors:** Peixian Luan, Tangbin Huo, Bo Ma, Dan Song, Xiaofeng Zhang, Guo Hu

**Affiliations:** ^1^ Heilongjiang River Fisheries Research Institute Chinese Academy of Fishery Sciences Harbin China; ^2^ Key Laboratory of Freshwater Aquatic Biotechnology and Breeding Ministry of Agriculture and Rural Affairs Heilongjiang River Fisheries Research Institute, Chinese Academy of Fishery Sciences Harbin China

**Keywords:** coancestry coefficient, effective population size, *Esox Lucius*, gene flow, identity by descent, Inbreeding

## Abstract

Northern pike (*Esox lucius*) was widely distributed in the high latitudes of the northern hemisphere. In China, northern pike was originally distributed only in the upper reaches of the Irtysh River in Xinjiang and has appeared in many water bodies outside the Irtysh River Basin in Northern Xinjiang. A total of four populations were collected from north to south in Xinjiang, including Irtysh River (RIR), Ulungu Lake (LUL), a small lake nearby Ulungu River (LJD), and Bosten Lake (LBO). We estimated population genomic parameters, performed gene flow analysis, and estimated the effective population size of each population. The proportion of individuals with high inbreeding coefficient (*F* ≥ 0.0625) accounted for 36.4% (44/121) of all sequenced individuals, approximately 4.5% (1/22) in LUL, 25.9% (7/27) in LBO, 42.9% (18/42) in RIR, and 60% (18/30) in LJD. RIR had the highest mean of genomic relatedness (coancestry coefficient = 0.025 ± 0.040, IBD = 0.036 ± 0.078). Gene flow results showed that the population spreading was from RIR into two branches, one was LBO, and the other continued to split into LUL and LJD, and migration signal from LBO to LUL was detected. Our results suggested that the extinction risk of northern pike was very low in Xinjiang of China, and the controlled capture fishery of northern pike could be developed reasonably.

## Graphical abstract

Genomic pairwise relatedness of individual pairs within or between populations from the four sampling populations can be identified by coancestry coefficient and IBD, and validated by the consistency of the two parameters. Heat maps for estimates of the genomic pairwise coancestry coefficient and IBD analyses are shown in Figure 4 (coancestry coefficient in Figure 4a, IBD in Figure 4b). Individual pairs within RIR population had the highest mean (*p* < .05) of genomic relatedness (coancestry coefficient = 0.025 ± 0.040, IBD = 0.036 ± 0.078), and individual pairs within LUL and LJD populations had relatively distant genomic relatedness (*p* < .05).

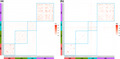

## 1 INTRODUCTION

Northern pike (*Esox lucius*) is an aggressive carnivorous fish that lives at the top of the food chain, known as “aquatic wolf,” and widely distributed in the high latitudes of the northern hemisphere (mainly above latitude 40° to the Arctic zone) (Forsman et al., 2015). Northern pike is known for their apparently low genetic variability, compared to other freshwater fishes (Pedreschi et al., 2014; Senanan & Kapuscinski, 2000). It was suggested as a reason of low genetic diversity for northern pike that the refuge of pike was greatly compressed northward during the glacial period in geological history, causing population bottleneck (Wooller et al., 2015). In the postglacial period, the fish population and distribution area expanded again, which resulted in a mismatch between genetic diversity and distribution area. Interestingly, northern pike has a strong ability to spread and colonize in changing environments, which is not commensurate with its low genetic diversity (Jacobsen et al., 2005; Miller & Kapuscinski, 1997).

In China, northern pike was originally distributed only in the upper reaches of the Irtysh River in the Xinjiang Uygur Autonomous Region (referred to as “Xinjiang”) (Huo et al., 2009). Xinjiang was located in the inland region of Central Asia where water resources were scarce, covering an area of 1.66 million square kilometers, accounting for about 1/6 of land area of China. Before the 1960s in China, the output of northern pike in the Irtysh River Basin accounted for about 20% of the total weight of the fish capture, and the annual output could reach 120 tons (Ren, 2002). In recent years, due to the drop‐off of water level in the Irtysh River, the deterioration of breeding conditions, and illegal overfishing, the pike population in the Irtysh River has declined sharply, and the capture output in 2006 was about 6% of the peak in the 1960s (Huo et al., 2009). At present, commercial fisheries have been completely banned in the Irtysh River Basin.

Similar to other Central Asian regions, due to lack of water resource, many water conservancies projects have been built to divert water from natural rivers to urban and rural areas in Xinjiang (Li & Su, 2008). These projects provide opportunities for fish to spread to new habitats, and northern pike has spread to the Ulungu Lake and its surrounding small rivers, lakes, and swamps outside the Irtysh River Basin through these channels, too. According to the monitoring of our institute, northern pike has appeared in the water system outside the Irtysh River since the 1980s (Jiang & Huo, 2014). Meanwhile, pike game fishing and consumption is an increasingly popular pastime in Xinjiang. With the continuous arrival of tourists, the consumption demand for northern pike is increasing (Yang et al., 2018). It becomes increasingly difficult to control the range and distance of illegally and disorderly spreading. As a result, northern pike has appeared in many water bodies outside the Irtysh River Basin in Northern Xinjiang and Bosten Lake in Southern Xinjiang with human activities (Guo et al., 2005). However, the number, distribution, and source of northern pike outside the Irtysh River Basin remain unknown.

The present study aimed to estimate population genomic parameters such as the genetic diversity, population structure, and connectivity of the various populations and to perform gene flow analysis among four population of northern pike captured from four habitats, including the Irtysh River, Ulungu Lake, a small lake nearby Ulungu River, and Bosten Lake. The methods mentioned above were performed to understand the genomic inbreeding and population structure of northern pike, help us trace the spread pattern better, and inform the management and conservation of northern pike fishery resources in Xinjiang, China.

## 2 MATERIAL AND METHODS

### 2.1 Water systems involved in sample collection

**FIGURE 1:**
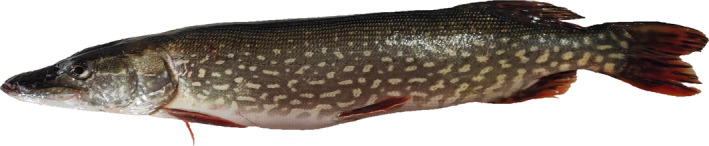
Wild fish of northern pike (*Esox lucius*) photographed by Peixian Luan with permission

**FIGURE 2:**
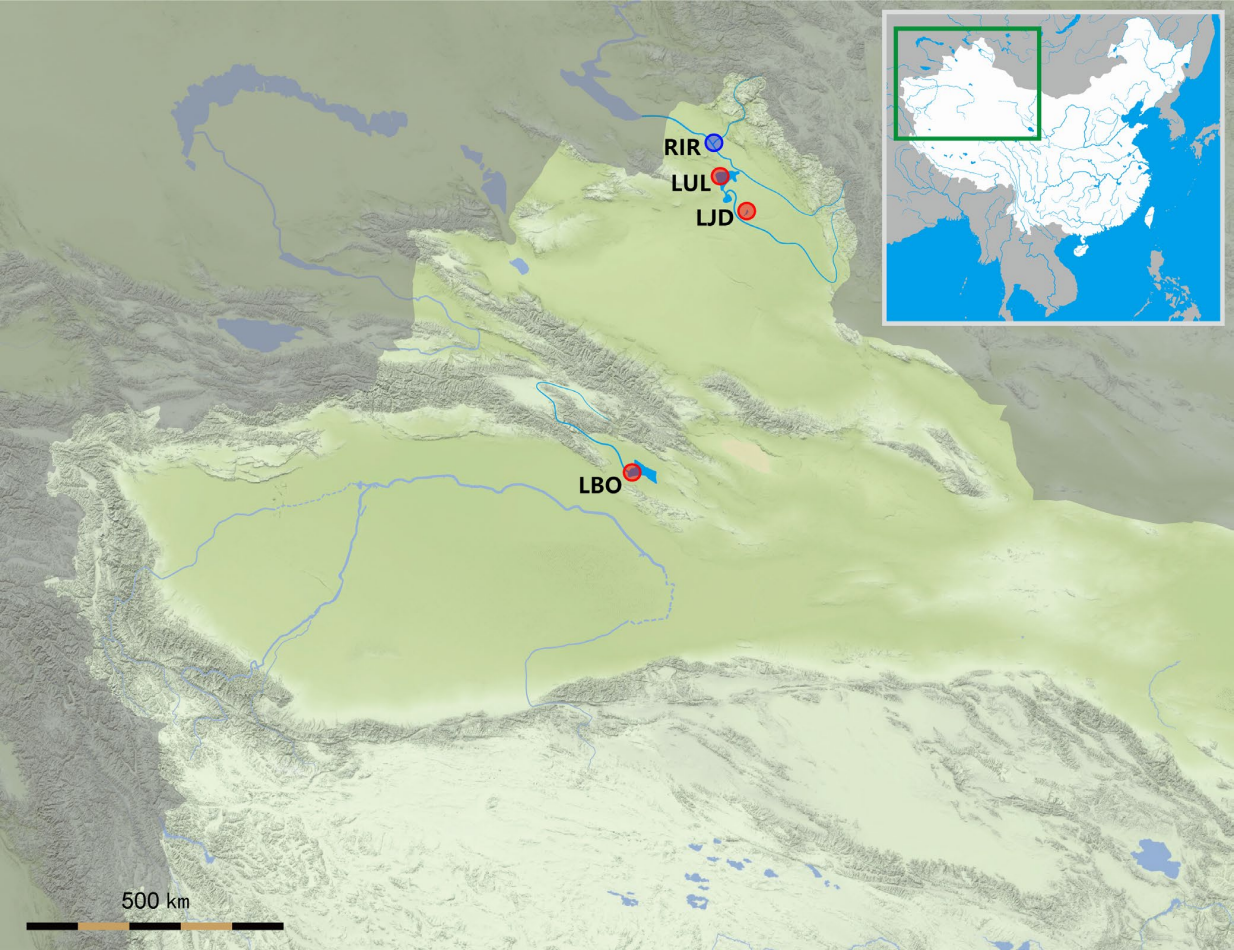
Four water systems involved in sample collection in this study. A total of four populations were collected from north to south in Xinjiang, China. Irtysh River population (RIR, 47°42'N, 86°50'W), Ulungu Lake population (LUL, 47°18'N, 87°02'W), the population of a small lake nearby Ulungu River (LJD, 46°41'N, 87°57'W), Bosten lake population (LBO, 41°50'N, 86°36'W). The blue circle represents a natural population. The red circle represents a current population

In this study, a total of four water systems were involved in sample collection of northern pike (Figure 1) from north to south in Xinjiang, including Irtysh River, Ulungu Lake, a small lake nearby Ulungu River, and Bosten Lake. These four water systems are abbreviated as RIR (Irtysh River), LUL (Ulungu Lake), LJD (a small lake nearby Ulungu River has no official name and near a small village called Jidiele), and LBO (Bosten Lake). The spatial distribution of the four water systems was shown on the map (Figure 2). Northern and Southern Xinjiang are separated by the Tianshan Mountains, and there is no waterway that crosses the Tianshan Mountains from Northern to Southern Xinjiang.

The Irtysh River is the only river that flows into the Arctic Ocean in China, and the Chinese section is about 546 kilometers of a total length of 4,248 kilometers (Jiang & Huo, 2014; Ren, 2002). Ulungu Lake is composed of two connected freshwater lakes, the Jili Lake with an area of 165 square kilometers and the Brento Sea with an area of 730 square kilometers, and the average depth of Ulungu lake is 8 meters (Jiang & Huo, 2014). Bosten Lake is the largest inland freshwater lake in Xinjiang, which is located in Southern Xinjiang and on the northeastern edge of the Tarim Basin (Guo et al., 2005). Bosten Lake is nearly 1,200 square kilometers, and the average depth of the lake is 9 m.

### 2.2 Animal ethics statement

The wild fish capture involved in this study had obtained a scientific fishing license granted by the Department of Agriculture and Rural Affairs of Xinjiang Uygur Autonomous Region. The fishing license must be renewed and re‐approved annually. All animal procedures in this study were conducted according to the guidelines for the care and use of laboratory animals of Heilongjiang River Fisheries Research Institute, Chinese Academy of Fishery Sciences (CAFS). The studies in animals were reviewed and approved by the Committee for the Welfare and Ethics of Laboratory Animals of Heilongjiang River Fisheries Research Institute, CAFS (Approval code: 2019‐03‐15).

### 2.3 Sampling populations

The wild fish were captured with standard gillnetting procedures. The standard gillnet was a sinking gillnet, 30 meters long and 3 meters high. The net had 3 mesh panels, each of which was 10 meters long. The mesh sizes were 10 cm, 12 cm, and 14 cm. RIR population (42 individuals) were captured from the mainstream of Irtysh River (47°42'N, 86°50'W) in August 2019. LUL population (22 individuals) were captured from Ulungu Lake (47°18'N, 87°02'W) in May 2019. LJD population (30 individuals) were captured in a small lake nearby Ulungu River (46°41'N, 87°57'W) in October 2019. LBO population (27 individuals) were captured from Bosten lake (41°50'N, 86°36'W) in September 2019. The 2‐phenoxyethanol (C_8_H_10_O_2_) was used as an anesthetic to relieve the pain of the fish during handling and cutting a small piece of caudal fins. Then, fin samples were stored in absolute ethanol in a refrigerator at −20℃ until the extraction of genomic DNA.

### 2.4 Genomic DNA extraction and SLAF sequencing

All fin samples were digested overnight with proteinase K, and genomic DNA was extracted using standard phenol–chloroform protocol (Psifidi et al., 2015). A NanoDrop 2000C spectrophotometer (Thermo Scientific) was used to detect the quality of DNA, and high‐quality genomic DNA was used to construct the specific locus amplified fragment (SLAF) library (Sun et al., 2013). For SLAF sequencing, the extracted genomic DNA was digested with the restriction enzymes RsaI and HaeIII. The gel extraction kit (Qiagen) was used to isolate DNA fragments with a size of 550–600 bp (with indexes and adaptors). Based on the reference genome sequence of *Esox lucius* at NCBI (Genome accession number: GCA_004634155.1, released by University of Victoria in April 2019), it was predicted that there were about 138,000 SLAFs. Paired‐end library construction was conducted as described in the Illumina sample preparation guide. All the 121 samples were sequenced on an Illumina HiSeq ×10 platform Genome Analyzer with a 2 × 126 bp setup (Illumina Inc.), according to the manufacturer's recommendations at the Experimental Department of Biomarker Technologies Corporation.

### 2.5 SNP calling

The algorithm of BWA‐MEM in the BWA software (Li & Durbin, 2010) was used to compare the SLAF‐seq reads with the reference genome of *Esox lucius* at NCBI (Genome accession number: GCA_011004845.1, released by Vertebrate Genomes Project on March 2020), with the settings (1 for score for a sequence match, 4 for penalty for a mismatch, default for other settings). The SelectVariants and MarkDuplicates tool of GATK (Mckenna et al., 2010) and the mpileup utility of SAMtools (Li et al., 2009) were used for SNP calling with default settings. Vcftools was used to filter SNPs meeting the following criteria (Danecek et al., 2011): SNPs calling rate across all samples ≥95%, quality scores (Quality Score for Illumina Next‐Generation sequencing) ≥ 30, minor allele frequency (MAF) ≥ 5%, the significance level of Hardy–Weinberg equilibrium (HWE) test ≥ 0.01, mean depth ≥ 100 reads, and only one SNP per SLAF. In total, 348.96 M clean paired‐end reads were generated across the 121 individuals after filtering. On average, 2.88 M paired‐end reads were obtained for each individual. The average Q30 was 92.68%, and the average GC content was 42.29%. A total of 1.52 M SLAFs were detected, in which 0.41 M SLAFs were polymorphic, and the average sequencing depth of these SLAFs was 7.48‐fold. Finally, 888,560 SNPs were obtained after SNP calling, among which there were 14,124 polymorphic SNPs passed the criteria and used in the genomic analysis in this study.

### 2.6 Genomic diversity and differentiation

The average observed heterozygosity (Ho) and the average expected heterozygosity (He) were estimated within population by using the R package “diveRsity” (Keenan et al., 2013). The Ho and He values of all 14,124 SNPs were calculated one by one within population, and then, the average value was taken as the Ho and He values of the population. The nucleotide diversity (Pi) was measured by SNPs in 10Mb windows across the entire genome using VCFtools (Danecek et al., 2011), and 2Mb steps were used to specify the step of size between windows. The average Pi of each population was calculated by mean of Pi value of all windows. Scheffe test was applied for the multiple comparisons of the means of the four populations using the R software (R Development Core Team, 2019). Pairwise *F*_ST_ values (Weir & Cockerham, 1984) were assessed via SNPs in a 10 Mb sliding window with a 2 Mb step size across the entire genome using VCFtools (Danecek et al., 2011), and the significance test of the paired *F*_ST_ was performed using permutation test (10,000 permutations).

### 2.7 Genomic relatedness and inbreeding

In this study, two parameters, including genomic coancestry coefficient and identity by descent (IBD), were used to measure the genomic relatedness between any two individuals. The genomic coancestry coefficient between two individuals was estimated based on the half off‐diagonal value in the genomic additive relationship, which were obtained using a mixed model based on the quantitative genetics model (Falconer & Mackay, 1996; Wang & Da, 2014). The formulation of genomic additive relationship was obtained by the genomic covariance, which can be expressed as following equation: $Ga=M_{\alpha }V_{a}M_{a}^{T}/m$ where a represented a total of additive effect, α was a vector of additive effects of SNP markers, *M_α_* was model matrix of α, *G_a_* was genomic additive relationship matrix, *V_a_* was covariance matrix of genotypes between two individuals, and m was the number of SNP markers. *G_a_* was calculated using a definition of genomic correlation proposed by Da et al. (2014), which assumed equal SNP variances across SNP markers and was a within‐SNP standardization of each SNP using its own SNP variance. The genomic coancestry coefficient between two individuals was estimated based on the half off‐diagonal value in *G_a_*, being the Definitions VI, implemented by GCORRMX program in the GVCBLUP software using genomic information (Wang et al., 2014). A hidden Markov model method was implemented for estimating genomic IBD between two individuals, assuming independence between the loci, and the standard method and default settings suggested in the PLINK documentation were used in the present study (Purcell et al., 2007). The individual's genomic inbreeding coefficient (*F*) was estimated by observed and expected autosomal homozygous genotype counts for each individual via PLINK software (Purcell et al., 2007). Multiple comparisons were also implemented by using Scheffe test for population means of genomic relatedness and inbreeding coefficients.

### 2.8 Population stratification and genetic structure

The genetic relationship matrix was extracted with the default parameters by principal component analysis (PCA) method using PLINK software (Purcell et al., 2007), and the first three feature eigenvectors were retained to create a two‐dimensional plot. The genetic structure of the 121 individuals was analyzed with the genome‐wide SNP markers via ADMIXTURE software (Alexander et al., 2009). Allele frequencies in the ancestral populations derived from multi‐locus genotype frequencies, setting a number of clusters (K) ranging from 1 to 10 with 10,000 iterations, and ADMIXTURE’s cross‐validation with 5‐fold were used to choose the correct value for K.

### 2.9 Gene flow and effective population size

Gaussian approximation was used to find genetic drift from genome‐wide allele frequency data and then to infer gene flows of historical population splits and mixtures for the four populations of northern pike by using Treemix software (Pickrell & Pritchard, 2012a, 2012b). Population history was modeled from zero to four migration events (0 ≤ *m* ≤ 4), and the block size was set to 100 and calculated the proportion of variance correlated between populations explained by each model. The tree was visualized using the R script embedded in Treemix.

The effective population size (Ne) can be inferred by the linkage disequilibrium model, which requires accurate estimation of linkage disequilibrium (LD) statistics from sequencing data. For two loci, LD is typically given by the covariance or correlation of alleles co‐occurring on a haplotype. Allele A has frequency p in the population (allele a has frequency 1−p), and B has frequency q (b has frequency 1−q). The covariance is denoted D, $D=\text{Cov}\left(\text{A,B}\right)=f_{\mathrm{AB}}‐\text{pq}=f_{\mathrm{ab}}‐f_{\mathrm{Ab}}f_{\mathrm{aB}}$. An extended approach for unbiased estimators for a large set of two‐locus statistics including *D* ^2^ and $\sigma_{D}^{2}$ was used to accurately estimate LD and calculate effective population size by Moments software (Ragsdale & Gravel, 2019). At the same time, a bias‐corrected version of the LD method was also used to infer the Ne, implemented with NeEstimator software (Do et al., 2014; Waples & Do, 2008). The 95% confidence intervals for point estimates were computed using 200 resampled bootstrap replicates in Moments and the parametric method in NeEstimator. Physical linkage was also taken into account when calculating Ne. In order to ensure that the locus used for LD analysis was located on a separate chromosome, so as not to underestimate Ne, an approach proposed by Waples et al. (2016) was applied to correct this bias, and the formulation was expressed as:

$$\text{Corrected}\;\text{Ne =}\;\frac{\text{Naive}\;\text{Ne}}{\left[0.098+0.219\times \ln\left(\text{chr}\right)\right]}.$$

## 3 RESULTS

### 3.1 Genetic diversity analysis for the populations

The value of Ho within each sampling population ranged from 0.299 to 0.323 (Table 1). The highest diversity was observed in LUL population, and the lowest diversity was found in LJD population. The results of multiple comparisons showed that there were significant differences in Ho of the four populations (*p* < .05). The value of He ranged from 0.314 to 0.317, and the highest diversity was observed in LUL and LJD populations, and the lowest diversity was found in RIR population (*p* < .05). The ranking of Pi results was similar to that of He, but the differences between populations were not significant (*p* > .05) in multiple comparisons; the detailed information is shown in Table 1.

**TABLE 1:** Summary statistics of genetic diversity for northern pike among 4 sampling populations in Xinjiang, China

Population	Sample size	Ho (mean ± *SD*)	He (mean ± *SD*)	Pi (mean ± *SD*)
RIR	42	0.306^a^ ± 0.161	0.311^a^ ± 0.150	4.17 × 10−6^a^ ± 1.84 × 10^–6^
LUL	22	0.323^b^ ± 0.176	0.317^b^ ± 0.151	4.26 × 10−6^a^ ± 1.85 × 10^–6^
LJD	30	0.299^c^ ± 0.157	0.317^b^ ± 0.148	4.23 × 10−6^a^ ± 1.83 × 10^–6^
LBO	27	0.313^d^ ± 0.175	0.314^ab^ ± 0.154	4.22 × 10−6^a^ ± 1.81 × 10^–6^

Notes

Within a column with no common lowercase superscript (a, b, c, and d) differs significantly in multiple comparisons (*p* < .05). Sampling populations: RIR (Irtysh River), LUL (Ulungu Lake), LJD (a small lake nearby Ulungu River), LBO (Bosten Lake).

Abbreviations: He, expected heterozygosity; Ho, observed heterozygosity; Pi, nucleotide diversity; *SD*, standard deviation.

### 3.2 Genomic inbreeding level estimates for the individuals

The average genomic inbreeding coefficients ($\bar{F}$) estimated from genome‐wide SNP data were 0.083 for LJD, 0.063 for RIR, 0.039 for LBO, and 0.012 for LUL, respectively (Table 2). Among them, the inbreeding level of the LJD population and the RIR population was very high, and the average value was higher than the value of the first‐level cousin mating (*F* ≥ 0.0625). The value of inbreeding coefficient for the LUL population was $\bar{F}$ = 0.012 ± 0.064, which was significantly lower than that of the other three populations in multiple comparisons (*p* < .05) (Table 2).

**TABLE 2:** Average genomic inbreeding coefficients, pairwise relatedness by population

Population	Sample size	Genomic inbreeding coefficients	Genomic pairwise relatedness	
		Mean ± *SD*	CC ± *SD*	IBD ± *SD*
RIR	42	0.063^ab^ ± 0.083	0.025^a^ ± 0.040	0.036^a^ ± 0.078
LUL	22	0.012^b^ ± 0.064	0.013^b^ ± 0.011	0.024^ab^ ± 0.039
LJD	30	0.083^a^ ± 0.055	0.011^b^ ± 0.017	0.004^c^ ± 0.024
LBO	27	0.039^ab^ ± 0.048	0.025^a^ ± 0.031	0.017^b^ ± 0.066

Notes

Within a column with no common lowercase superscript (a, b, and c) differs significantly in multiple comparisons (*p* < .05). Sampling populations: RIR (Irtysh River), LUL (Ulungu Lake), LJD (a small lake nearby Ulungu River), LBO (Bosten Lake).

Abbreviations: CC, genomic pairwise coancestry coefficients; IBD, probability of identify by descent; *SD*, standard deviation.

**FIGURE 3:**
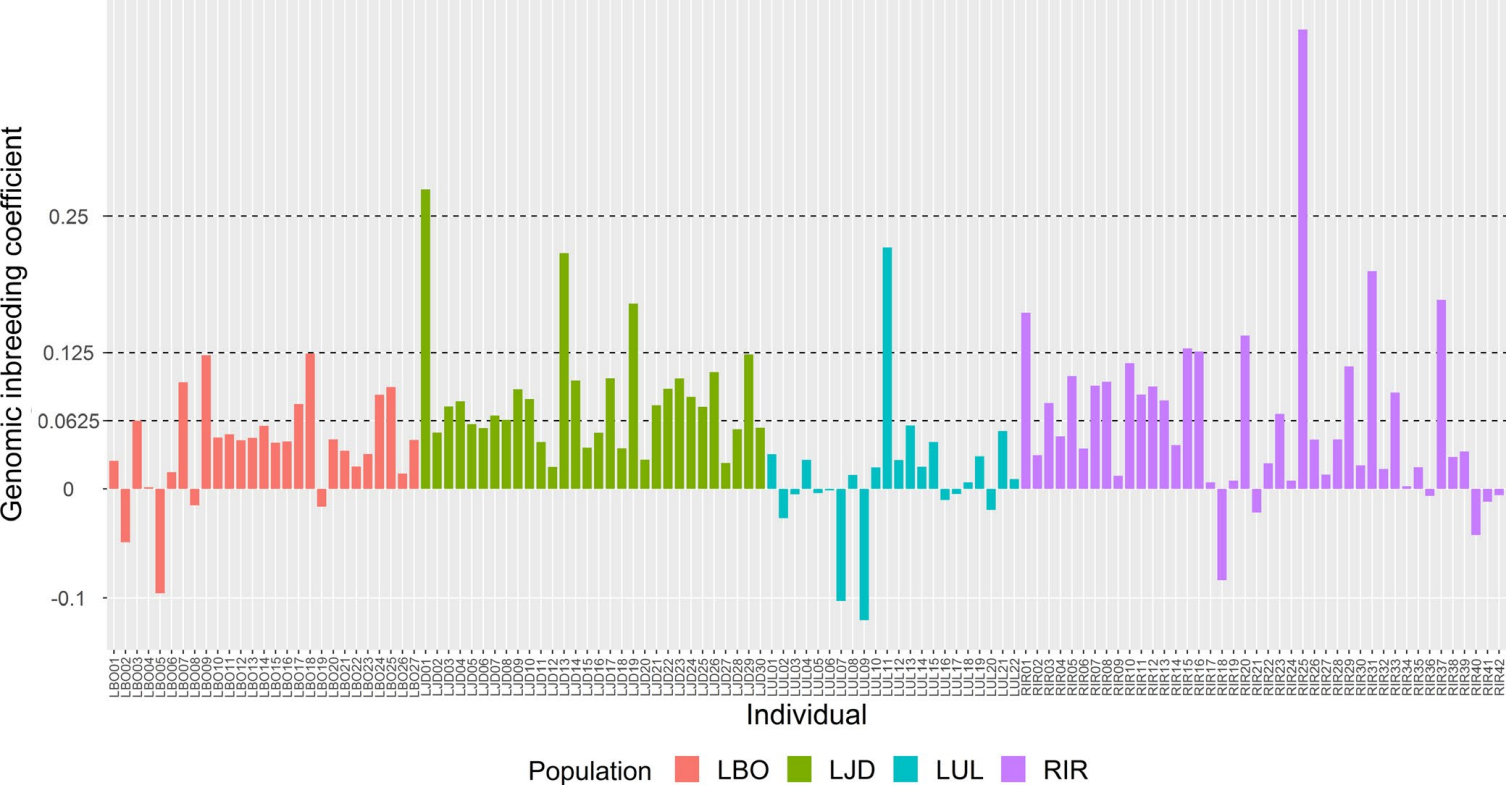
Genomic inbreeding coefficients of the 121 sequenced individuals of four populations. The genomic inbreeding coefficient (*F*) of each individual was estimated by observed and expected autosomal homozygous genotype counts for each individual via PLINK software (Purcell et al., 2007). Sampling populations: RIR (Irtysh River), LUL (Ulungu Lake), LJD (a small lake nearby Ulungu River), LBO (Bosten Lake)

The proportion of individuals with an inbreeding coefficient greater than 0.0625 accounted for 36.4% (44/121) of all individuals (Figure 3), approximately 4.5% (1/22) in LUL population, 25.9% (7/27) in LBO population, 42.9% (18/42) in RIR population, and 60% (18/30) in LJD population. The number of individuals with inbreeding coefficient greater than 0.125 (the level reached by half‐sib mating) was 11, including 1 in LUL population, 3 in LJD population, and 7 in RIR population. The number of individuals greater than 0.25 (the level reached by full sibling mating) was 2, including one in RIR population and the other one in LJD population.

### 3.3 Genomic pairwise relatedness within and between populations

**FIGURE 4:**
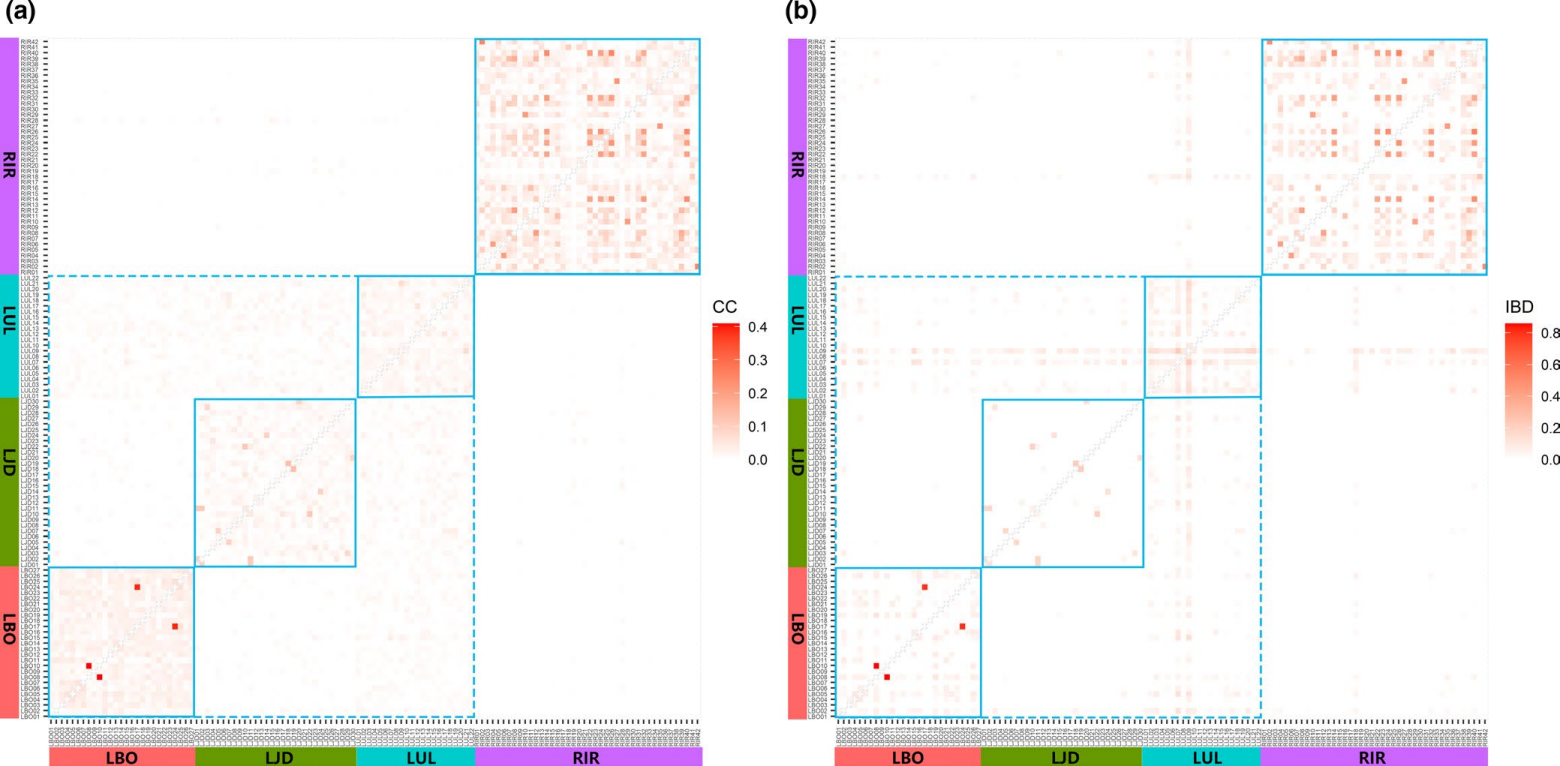
The overview diagram of genomic pairwise coancestry coefficients and probability of identical by descent (IBD) between the 121 sequenced individuals. (a) Genomic pairwise coancestry coefficients (CC). (b) Probability of identical by descent (IBD). Sampling populations: RIR (Irtysh River), LUL (Ulungu Lake), LJD (a small lake nearby Ulungu River), LBO (Bosten Lake)

Genomic pairwise relatedness of individuals pairs within or between populations from the four sampling populations can be identified by coancestry coefficient and IBD, and validated by the consistency of the two parameters. Heat maps for estimates of the genomic pairwise coancestry coefficient and IBD analyses are shown in Figure 4 (coancestry coefficient in Figure 4a, IBD in Figure 4b), and the average genomic pairwise relatedness within each of four populations are shown in Table 2. Individual pairs within RIR population had the highest mean (*p* < .05) of genomic relatedness (coancestry coefficient = 0.025 ± 0.040, IBD = 0.036 ± 0.078), and individual pairs within LUL and LJD populations had relatively distant genomic relatedness (*p* < .05).

According to the relationship inference criteria based on the coancestry coefficient (Figure 4a), 3 pairs (1 in RIR and 2 in LBO) were inferred to be full‐sibs or closer relatives (coancestry coefficient ≥ 0.25), 28 pairs (25 in RIR,1 in LJD and 2 in LBO) to be half‐sibs or closer relatives (0.125 ≤ coancestry coefficient < 0.25), and 106 pairs (92 in RIR,1 in LUL, 9 in LJD, and 4 in LBO) to be first cousins or closer relatives (0.0625 ≤ coancestry coefficient < 0.125). At the same time, no pairs of close relatives inter populations were detected. The average values of genomic coancestry coefficient between different populations were all negligible values close to zero, and some were negative. The IBD results were similar to those of the coancestry coefficient analysis (Figure 4b), except that no negative values appeared (Table 3).

**TABLE 3:** Average genomic pairwise relatedness between different populations

Between different populations		Genomic pairwise relatedness	
Population A	Population B	CC ± *SD*	IBD ± *SD*
RIR	LUL	−0.0208 ± 0.0098	0.0030 ± 0.0135
RIR	LBO	−0.0224 ± 0.0101	0.0006 ± 0.0046
RIR	LJD	−0.0159 ± 0.0096	0.0001 ± 0.0016
LUL	LBO	0.0013 ± 0.0086	0.0058 ± 0.0184
LUL	LJD	0.0023 ± 0.0095	0.0047 ± 0.0155
LBO	LJD	−0.0077 ± 0.0084	0.0003 ± 0.0037

Notes

Sampling populations: RIR (Irtysh River), LUL (Ulungu Lake), LJD (a small lake nearby Ulungu River), LBO (Bosten Lake).

Abbreviations: CC, genomic pairwise coancestry coefficients; IBD, probability of identify by descent; *SD*, standard deviation.

### 3.4 Population stratification and genetic structure analysis

**FIGURE 5:**
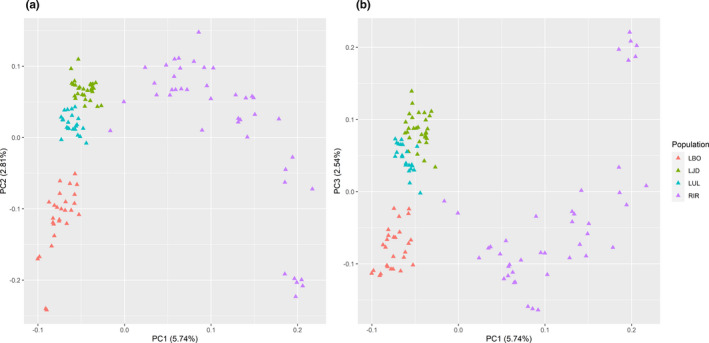
Principal component analysis (PCA) of 121 sequenced individuals. (a) Two‐dimensional plot of the first principal component and the second principal component; (b) two‐dimensional plot of the first principal component and the third principal component. Sampling populations: RIR (Irtysh River), LUL (Ulungu Lake), LJD (a small lake nearby Ulungu River), LBO (Bosten Lake)

The pairwise *F*_ST_ between any two of the four populations ranged from 0.008 to 0.051, showing low genetic differentiation (Table 4). Among them, the *F*_ST_ between RIR and LBO was of the largest value (*F*_ST_ = 0.051). Population stratification was detected by PCA method. The 2D graphs for the first three PCs of these 121 individuals showed that the four populations were stratified, and only LJD and LUL showed a little bit overlapped. Among them, the RIR population had the highest genomic diversity (Figure 5).

**TABLE 4:** Pairwise *F*_ST_ between populations

	RIR	LUL	LJD	LBO
RIR	–	0.041	0.037	0.051
LUL	–	–	0.008	0.020
LJD	–	–	–	0.027
LBO	–	–	–	–

Notes

Sampling populations: RIR (Irtysh River), LUL (Ulungu Lake), LJD (a small lake nearby Ulungu River), LBO (Bosten Lake). *p* < .0001 for all comparisons.

Abbreviation: *F*_ST_, the fixation index for genetic differentiation.

**FIGURE 6:**
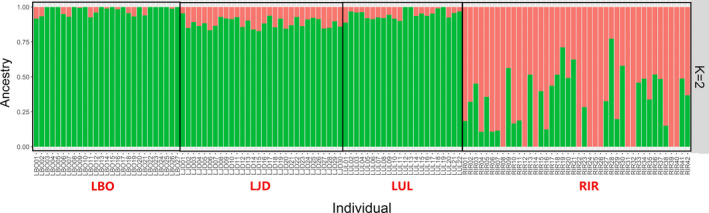
Genetic structure of the 121 sequenced individuals based on *K* = 2 using ADMIXTURE. Each column represents a single individual, and the proportion of the colored part represents the estimated genetic ancestry per individual. Sampling populations: RIR (Irtysh River), LUL (Ulungu Lake), LJD (a small lake nearby Ulungu River), LBO (Bosten Lake)

All the 14,124 SNPs were used to detect the genetic structure for the 121 individuals. The cross‐validation results of genetic structure analysis suggested *K* = 2 with the lowest probability of errors (Result shown in supplementary materials). Analysis of the genetic structure suggested that the RIR population contained the genetic information of two presumed ancestor populations, while the other three populations were dominated by the same one ancestor population (*K* = 2) (Figure 6).

### 3.5 Gene flow and spread history analysis

**FIGURE 7:**
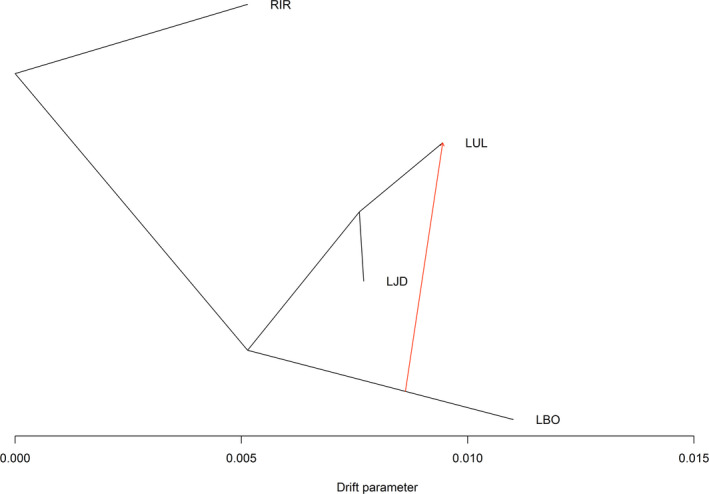
Gene flow analysis and spread history inferring for the four populations. The black line indicates the pattern of population spread, and the red line with arrows indicates the inferred migration direction (Migration weight = 0.25). Sampling populations: RIR (Irtysh River), LUL (Ulungu Lake), LJD (a small lake nearby Ulungu River), LBO (Bosten Lake)

Gene flow results showed that the population splitting was from the RIR population into two branches, one was the LBO population, and the other continued to split into the LUL and LJD populations (Figure 7). When Treemix sets the migration from 0 to 4, the topology of the tree was the same, and only one migration signal from LBO to LUL appeared for all of migration from 1 to 4. Regarding gene flow, our results support migration signal from the LBO to LUL population (m = 1).

### 3.6 Effective population size estimates

We used two different software (Moments and NeEstimator) to estimate Ne of the four populations and obtained similar results. The ranking of Ne estimates for all four populations by these two methods was the same. However, the LUL estimate obtained by Moments was much higher than the value estimated by NeEstimator. Based on the number of haploid chromosomes (chr = 25), the Ne correction factor was approximately equal to 1.245. LUL had the largest Ne (1,101.7 using Moments, and 824.9 using NeEstimator), LJD was the second largest Ne (263.4 using Moments, and 310.1 using NeEstimator), LBO was the third largest Ne (91.8 using Moments, and 93.0 using NeEstimator), and RIR was the smallest Ne (67.5 using Moments, and 72.7 using NeEstimator) (Table 5).

**TABLE 5:** Corrected effective population sizes for each population of northern pike

Population	Ne using moments	Ne using Ne estimator
RIR	67.5	72.7
LUL	1,101.7	824.9
LJD	263.4	310.1
LBO	91.8	93.0

Note

Ne, the effective population size. Sampling populations: RIR (Irtysh River), LUL (Ulungu Lake), LJD (a small lake nearby Ulungu River), LBO (Bosten Lake). The results of naive Ne estimates directly inferred by the software were shown in the supplementary materials.

## 4 DISCUSSION

Northern pike is widely distributed and have strong adaptability to various ecological types of habitats (Nordahl et al., 2019). However, it is interesting that there is a low genetic diversity among the populations (Bekkevold et al., 2015; Wennerström et al., 2016). The commonly used genetic diversity parameters consistently show low genetic diversity in northern pike, whether based on the results of low‐density markers (such as isozyme, mitochondrial DNA, microsatellite), or high‐density SNP markers (Pierce, 2012; Sunde et al., 2020). Our results showed that the Ho value of these pike populations in Xinjiang ranged from 0.299 to 0.323, which was higher than North American (0.110) and Siberian pike populations (0.140) (Senanan & Kapuscinski, 2000), which was comparable to Sweden pike populations (0.208–0.289) (Sunde et al., 2020), but it was obviously lower than the average Ho of 0.460 for freshwater fish in previous reports (Dewoody & Avise, 2000). In some water‐scarce areas of Xinjiang, northern pike was particularly vulnerable to loss genetic diversity. Meanwhile, freshwater fish often suffer from habitat loss or fragmentation, small population mating, and illegal overfishing (Closs et al., 2015). Moreover, as the top predator, northern pike usually was the largest and most successful spawner in a particular aquatic ecosystem in Xinjiang, and the offspring were also most likely to survive (Pedreschi et al., 2014; Pierce, 2012). As long as a few individuals successfully expand their habitats, they can establish new stable populations (Aguilar et al., 2005). Similarly, a small number of individuals undergo population expansion through a series of founder expansion activities, presumably resulting in a widespread lack of variability in the pike populations (Johnson, 2019).

For the level of inbreeding, there were a large number of individuals with high inbreeding coefficients in the pike populations in Xinjiang, especially the RIR and LJD populations, both of whose average population inbreeding coefficients surpassed the inbreeding level of first‐level cousin mating (*F* ≥ 0.0625). At the individual level, we found 42.9% (18/42) of the individuals in the RIR population were inbreeding offspring (*F* ≥ 0.0625). Comparatively, only LUL population had a lower inbreeding coefficient ($\bar{F}$ = 0.012 ± 0.064). At present, it was difficult to obtain direct evidence of the reasons for the high inbreeding level of the populations. Our results suggested that these populations might be suffered by high‐intensity capture fishing in the earlier period. In fact, this could also be partially inferred from the capture records in the Irtysh River, as the output in 2006 was only 6% of the peak in the 1960s (Huo et al., 2009). The collapse of the RIR population might lead to a rapid increase in the level of inbreeding. There were a large number of shallow wet areas in the lakes that could not be fished with nets, so more breeding individuals of the lake populations were retained.

The genomic coancestry coefficients and IBD of these four populations were all low, but coefficients of RIR and LBO were significantly higher than LUL and LJD. The low value of genomic coancestry coefficient and IBD for inter‐population between RIR and other three populations, along with PCA and genetic structure results visually displayed, RIR population was relatively distant from the other three populations. This structure was a bit confusing because it did not match the field observation records of population spread. In this respect, we performed demographic history analysis using Fastsimcoal software (Excoffier et al., 2013), and it did not seem to give the possibility of another source of the populations. The timing of the possible historical events did not match the fisheries observation data (results shown in supplementary materials). One possible explanation was that the high level of inbreeding had caused rapid genetic drift in RIR population, leading to major changes in the genetic structure, while the other three populations still maintained similar allele frequencies when the population was established. This result suggested that the genetic structure of the other three populations may be closer to the original population of the Irtysh River than the current RIR population.

Besides, the ADMIXTURE cross‐validation result showed that *K* = 2. When *K* = 2, researchers should pay attention to the substructures in the two branches (Janes et al., 2017). In order to prevent the RIR from being extremely different from the other three populations, the substructures of the three populations were compressed to form one group. We removed RIR and analyzed the genetic structure of the other three populations of LUL, LJD, and LBO. The results showed that no genetic structuring in or across these three populations (the results shown in the supplementary material).

In this study, there were some negative values in the genomic inbreeding coefficient and coancestry coefficient. In classical quantitative genetics, the estimated values of these two parameters would not appear negative. However, when using genomic information to estimate two genetic parameters above, sometimes negative values were generated. Usually, these small negative estimates were considered as small values close to zero or “unrelated” (Garbe et al., 2016). The reasons for these negative values could be attributed to the following two points. On the one hand, in terms of definition, a negative genomic inbreeding coefficient value should not be interpreted as a probability as in classical genetics, but it should be interpreted as a correlation, so the *F*‐estimation equation still applied (Powell et al., 2010). From the perspective of gamete IBD and identity by state (IBS), the average value of inbreeding coefficient between alleles in the current population was defined as 0, and the inbreeding coefficient between some pairs of gametes could be negative (Powell et al., 2010). One the other hand, the bias of the estimates might be caused by insufficient density and uniformity of SNP markers dataset obtained by simplified genome sequencing method such as SLAF‐seq (Ackerman et al., 2017; Sun et al., 2013; Zhou et al., 2016).

LUL had the largest effective population, which might be partially attributed to the diversion channels that allow individuals of RIR and surrounding small lakes to have the opportunity to migrate into LUL population. At the same time, gene flow results showed some individuals from LBO were also brought into LUL population. In the initial species record of China, northern pike had only been distributed in the Irtysh River, and there were no pike distribution in Bosten Lake in Southern Xinjiang. In the absence of waterway traffic between Northern and Southern Xinjiang, some pike individuals should be brought to Bosten Lake along with human activities and brought back to Ulungu Lake sometime after the fish successfully colonized and formed population. The Ne of LJD (about 300) was not commensurate with the size of the habitat. Under similar He and Ho estimates of the other populations (RIR, LUL, LBO), the high Ne of LJD population might be caused by multiple reasons, such as sampling bias, a large number of rare alleles, or even occurring sharply decline in population size due to habitat destruction or illegal fishing. Besides, this result partially supported our opinion that the northern pike populations in the water bodies surrounding Ulungu Lake might have connectivity with the LUL population by channels or other forms of waterways. One additional reason for the smallest estimated value of RIR was that the sample collected from the Irtysh River may not fully represent all fragmented habitats of the Irtysh River blocked by dams, which might underestimate the effective population size.

As an aggressive carnivorous species, northern pike might be very destructive to the invasive water ecosystem. The introduction of nonnative species posed an increasing threat to the functional structure of the original aquatic ecosystem and might cause many ecological problems, such as extinction of native species through competition, predation, introduction of new pathogens, hybridization with native species, and changes in nutritional niche structure of ecosystem (Aguilar et al., 2005; Bøhn & Amundsen, 2001; Ehrenfeld, 2010; Simberloff et al., 2013). In the conservation biology approach, invasive northern pike should be cleared from the lake ecosystems. However, given the fact that northern pike has been widely distributed and large effective population size in Xinjiang's natural water systems, our policy recommendation to the local fishery department is to rationally develop capture fisheries of northern pike under the condition of comprehensive consideration of commercial and ecological factors.

## 5 CONCLUSIONS

In summary, the evidence we obtained from this study suggests that the current wild populations of northern pike in Xinjiang should be originated from the Irtysh River. At present, a high level of inbreeding was found for the northern pike population in the Irtysh River, and the genomic relatedness among individuals within the population was also very close. Meanwhile, the northern pike in Xinjiang has established stable populations in many waters of Xinjiang outside the original habitat. In terms of conservation genetics, the extinction risk of northern pike was very low in Xinjiang of China. Under the conditions of evaluating the amount of fishery resources and the genetic structure of the populations, the controlled capture fishery of northern pike could be developed reasonably.

## CONFLICT OF INTEREST

None declared.

## AUTHOR CONTRIBUTIONS

**Peixian Luan:** Data curation (equal); Formal analysis (equal); Methodology (lead); Software (lead); Validation (lead); Visualization (lead); Writing‐original draft (supporting); Writing‐review & editing (supporting). **Tangbin Huo:** Data curation (equal); Formal analysis (equal); Resources (equal). **Bo Ma:** Data curation (supporting); Resources (supporting). **Dan Song:** Data curation (equal); Investigation (equal); Resources (equal); Writing‐original draft (supporting). **Xiaofeng Zhang:** Data curation (equal); Formal analysis (supporting); Investigation (supporting); Methodology (supporting); Software (supporting); Writing‐original draft (supporting). **Guo Hu:** Conceptualization (lead); Formal analysis (lead); Funding acquisition (lead); Investigation (lead); Methodology (lead); Project administration (lead); Resources (lead); Supervision (lead); Validation (lead); Writing‐original draft (lead); Writing‐review & editing (lead).

## Data Availability

All the raw sequence data were submitted to BIG database (The BIG Data Center at Beijing Institute of Genomics) and are publicly available (Accession numbers: PRJCA002958). All descriptive statistics for the sequencing dataset, as well as genetic parameter estimates of all individuals, genomic relatedness measures for each individual pair within and between the four populations, *p* values of statistical significance, and the original data related to the tables and figures in this article were stored in Dryad (https://doi.org/10.5061/dryad.hhmgqnkds), and the detailed information of the supplementary tables and figures was indexed in the README file.
